# Comparing Visually Assessed BI-RADS Breast Density and Automated Volumetric Breast Density Software: A Cross-Sectional Study in a Breast Cancer Screening Setting

**DOI:** 10.1371/journal.pone.0136667

**Published:** 2015-09-03

**Authors:** Daniëlle van der Waal, Gerard J. den Heeten, Ruud M. Pijnappel, Klaas H. Schuur, Johanna M. H. Timmers, André L. M. Verbeek, Mireille J. M. Broeders

**Affiliations:** 1 Radboud university medical center, Radboud Institute for Health Sciences, Nijmegen, The Netherlands; 2 Dutch Reference Centre for Screening, Nijmegen, The Netherlands; 3 Department of Radiology, Academic Medical Centre, University of Amsterdam, Amsterdam, The Netherlands; 4 Department of Radiology, University Medical Center Utrecht, Utrecht, The Netherlands; ISPO, ITALY

## Abstract

**Introduction:**

The objective of this study is to compare different methods for measuring breast density, both visual assessments and automated volumetric density, in a breast cancer screening setting. These measures could potentially be implemented in future screening programmes, in the context of personalised screening or screening evaluation.

**Materials and Methods:**

Digital mammographic exams (N = 992) of women participating in the Dutch breast cancer screening programme (age 50–75y) in 2013 were included. Breast density was measured in three different ways: BI-RADS density (5^th^ edition) and with two commercially available automated software programs (Quantra and Volpara volumetric density). BI-RADS density (ordinal scale) was assessed by three radiologists. Quantra (v1.3) and Volpara (v1.5.0) provide continuous estimates. Different comparison methods were used, including Bland-Altman plots and correlation coefficients (e.g., intraclass correlation coefficient [ICC]).

**Results:**

Based on the BI-RADS classification, 40.8% of the women had ‘heterogeneously or extremely dense’ breasts. The median volumetric percent density was 12.1% (IQR: 9.6–16.5) for Quantra, which was higher than the Volpara estimate (median 6.6%, IQR: 4.4–10.9). The mean difference between Quantra and Volpara was 5.19% (95% CI: 5.04–5.34) (ICC: 0.64). There was a clear increase in volumetric percent dense volume as BI-RADS density increased. The highest accuracy for predicting the presence of BI-RADS c+d (heterogeneously or extremely dense) was observed with a cut-off value of 8.0% for Volpara and 13.8% for Quantra.

**Conclusion:**

Although there was no perfect agreement, there appeared to be a strong association between all three measures. Both volumetric density measures seem to be usable in breast cancer screening programmes, provided that the required data flow can be realized.

## Introduction

Fibroglandular breast tissue, which is referred to as dense tissue, is known to mask breast carcinomas on mammograms [1, 2]. In addition to being a very strong independent breast cancer risk factor [2–4], high mammographic density is thus also associated with a decreased sensitivity of mammographic screening [2, 5]. Based on these associations, breast density could potentially be an important factor in breast cancer risk prediction and evaluation of breast cancer screening programmes. It might even become more important if considered for personalised screening [6]. Evidence on alternative screening regimens for population-based organized screening programmes is still limited, but additional screening modalities for women with a high breast density are extensively studied. Mammographic density can, however, only be used for evaluation or risk-stratified screening when it is assessed in an objective and reproducible manner.

Wolfe proposed a breast pattern scale in 1976 [7]. This led to the introduction of many other classifications in the following years, such as the Tabár scale [8] and the Breast Imaging Reporting and Data System (BI-RADS) density scale [9]. The latter is still used in breast cancer screening in the USA. A major drawback of these methods is the intra- and inter-rater variability [10]. More quantitative measures were therefore developed, with the area-based threshold software Cumulus ultimately becoming the standard method for breast density assessment in scientific research. Cumulus density values are, however, still subject to some measurement variability, and the use of the software within nationwide screening programmes is too time-consuming [11]. Furthermore, the introduction of digital mammography opened up a range of possibilities regarding automated methods that no longer assess dense area but dense volume. Dense volume, which takes breast thickness into account, is expected to be a more ‘biologically relevant’ measure [12, 13]. The commercial software programs Quantra and Volpara are now both commonly used, yet data on associations between these different methods is still scarce [14–17].

Breast density is not structurally assessed at screening examinations in the Netherlands. BI-RADS density is only recorded in the clinical setting. The Breast Density Inform Law in the USA [18] did lead to parliamentary questions in the Netherlands on the potential introduction of breast density measurements. With this increasing interest in breast density, it is important to find ways to obtain and report information on breast density of women participating in screening [19]. We thus have to learn more about the available methods. There is currently no consensus on what method to use for measuring breast density in the context of a screening programme. The objective of this study was therefore to compare different methods to measure breast density in the Dutch screening setting. The methods included here are BI-RADS density (visually assessed) and two volumetric software programs (Quantra and Volpara).

## Materials and Methods

### Setting

In the Netherlands, women ages 50–75 years are invited to participate in breast cancer screening every two years. We included 1000 mammographic examinations of participants who were screened at the Nijmegen screening unit in 2013. The dataset consists of multiple small sets of consecutive exams. The dates of retrieval were chosen at random, and we therefore believe that the dataset as a whole can be seen as a random sample of the Nijmegen screening population. Both a mediolateral oblique (MLO) and a craniocaudal (CC) view were obtained per breast. In five participants, only the left (N = 3) or the right (N = 2) views were available. Five examinations were excluded because the women had breast prostheses, which would distort the automated breast density measurements. In addition, three exams could not be read by the volumetric breast density software. This resulted in a dataset of 992 mammograms.

### Ethics statement

According to the Dutch law, medical ethics approval is not needed for this type of study, with no extra burden for participants and anonymized data. Written informed consent was not required for this study because the data were obtained in the context of an agreement between the regional screening organisations and the Dutch Reference Centre for Screening. Women automatically consent to the use of their data for scientific purposes by participating in screening. The screening organisations are responsible for data delivery in accordance with privacy regulations, particularly regarding anonymizing data and potentially removing data of participants who objected to the exchange of personal data with specific organisations (opt-out procedure).

### Breast density measurements

The Dutch screening programme uses Full-Field Digital Mammography (FFDM). All exams in this study were performed on the same Hologic Selenia system (Bedford, USA). Breast density was measured in three different ways: BI-RADS density (visually assessed by radiologists), Quantra volumetric density (automated software), and Volpara volumetric density (automated software). The R2 Quantra Volumetric Assessment software (version 1.3) was integrated in the Cenova DICOM server (version 2.1; Hologic, Bedford, USA). Version 1.5.0 of the Volpara Algorithm (Volpara Imaging Software 1.5.11; Mātakina, Wellington, New Zealand) was used.

BI-RADS breast density was assessed by three experienced screening radiologists. An initial pilot was performed where the radiologists scored the first 250 mammograms (from the original dataset of 1000 mammograms), which was concluded with a consensus meeting to ensure that the radiologists were applying the scale in a similar way. The ACR guidelines were discussed during the meeting, and discrepancies in the pilot scores were addressed. The consensus meeting had a favourable effect on the agreement between the radiologists. The scores before the consensus meeting were not included in our main analyses. Instead, the mammograms were scored again by the radiologists (individually) several weeks after the consensus meeting.

The overall scores were based on the agreement between at least two of the three radiologists. In the rare cases that all three radiologists disagreed (n = 9), the middle score was used. The mammograms were scored according to the newest (5^th^ edition, American College of Radiology) BI-RADS density classification [9]. In contrast to previous versions of the BI-RADS density classification, the qualitative categories are not matched to area-based density percentages in the new edition. The BI-RADS density categories in the 5^th^ edition are: (a) fatty, (b) scattered density, (c) heterogeneously dense, and (d) extremely dense [9]. A subset of 250 mammograms was scored twice by each radiologist to assess intra-observer variability. This was a different subset (mammogram 251–500) than the subset that was used in the pilot session. All assessments were performed on processed images at a review workstation. The radiologists were blinded to their previous scores and scores of others.

Quantra and Volpara are fully automated software programs that both assess the volumetric breast density on ‘for processing’ (raw) image data [17, 20, 21]. The X-rays are attenuated, as a result of photon absorption and scattering, in varying degrees as they pass through the different tissues. Estimates of fibroglandular tissue volume (absolute dense volume, in cm^3^) are based on the measured X-ray attenuation per pixel. Dividing the fibroglandular tissue volume by the total breast volume gives an estimate of the percentage volumetric breast density (percent dense volume). Volpara has developed an additional measure of breast density, namely the Volpara Density Grade (VDG). The VDG is based on percent dense volume, which is divided as follows: 0.0–4.5% (VDG1), 4.5–7.5% (VDG2), 7.5–15.5% (VDG3), and ≥15.5% (VDG4). The categories are based on agreement with the BI-RADS density scale.

### Statistical analyses

We present different agreement and reliability measurements to compare the density measurements [22]. Reliability refers to the ability to differentiate between women with a different density level [23]. Agreement, on the other hand, refers to the degree of similarity between two measurements. When two raters, for example, give different density values, the agreement between these measurements will be poor. Reliability can, however, still be substantial when the raters give the same women relatively low or high density scores. Agreement depends on measurement error, whereas with reliability measures the measurement error is related to the between-subject variability [23].

Weighted kappa scores (κ_w_; Fleiss-Cohen, quadratic weights) with corresponding 95% confidence intervals (CI) were used to assess the intra- and inter-rater reliability of the BI-RADS density scores [24]. The kappa scores were also compared to the categories originally defined by Landis and Koch [25] and slightly reworded by Altman [26]: poor (<0.20), fair (0.21–0.40), moderate (0.41–0.60), good (0.61–0.80), and very good (>0.80) reliability. In addition, we present the overall proportions of agreement (absolute agreement). This is the proportion of the scores that were exactly the same for two ratings.

The volumetric breast density estimates were compared to the BI-RADS classification by determining the median and the inter-quartile ranges (IQR) according to BI-RADS category for each volumetric density measure. We did not define a golden standard for breast density in our study. Receiver operating characteristic (ROC) analyses were, however, used to assess the ability of both volumetric software programs to differentiate between women with a high breast density (BI-RADS c+d) and women with a low breast density (BI-RADS a+b) based on the visual BI-RADS classification. This was done to enable comparisons with the literature. Chosen cut-off values were based on the highest accuracy, which we calculated using the following formula:

$$\frac{True-positives+True-negatives}{N}$$

In this study, ‘true-positives’ are women with a breast density of BI-RADS c+d who are classified as having a high breast density based on the volumetric estimates. ‘True-negatives’, on the other hand, refers to women with a BI-RADS a+b density who also have a low volumetric density.

The volumetric breast density measures were also compared to each other. Both Pearson’s correlation coefficients (r), based on log-transformed values (ln[x+1]), and two-way mixed intraclass correlation coefficients (ICC) were calculated for comparison of the different volumetric density measures. The following formula was used to calculate the ICC [23]:

$$ICC=\frac{\sigma_{p}^{2}}{\sigma_{p}^{2}+\sigma_{s}^{2}+\sigma_{res}^{2}}$$

$\sigma_{\text{p}}^{2}$ Variance as a result of differences between participants

$\sigma_{\text{s}}^{2}$ Variance as a result of differences between software programs

$\sigma_{\text{res}}^{2}$ Residual variance

An ICC of +1.0 indicates that the measures give perfectly matching scores, with ICC values >0.7 often being considered as ‘good’ [23, 27]. However, this cut-off point is rather arbitrary, and some have argued that the ICC should be at least 0.9 when measures have to be used interchangeably in clinical practice [22]. Confidence intervals were obtained by bootstrapping.

Finally, Bland-Altman plots are presented as agreement measures. The Bland-Altman plot consists of differences between two measurements on the y-axis and the mean of the two methods on the x-axis. Limits of agreement can be calculated by multiplying the standard deviation (σ) of the differences with 1.96 (+/-1.96σ). This is based on the assumptions that: (a) the variation in differences is similar across the range of values for the mean, and (b) the differences follow a normal distribution. The original (untransformed) differences were used for the Bland-Altman analyses. The observed difference between Quantra and Volpara is expected to be in between the limits of agreement in 95% of (future) measurements. Bias is defined as the mean difference between the two methods. The standard error of the bias is calculated as:

$$\sqrt{\frac{\sigma^{2}}{N}}$$

Age was the only other breast cancer risk factor available in this study population. As a descriptive analysis, the association between age and breast density was assessed by calculating proportions (BI-RADS density) and medians (Quantra and Volpara estimates) for each age group.

All statistical analyses were performed using SAS (version 9.2, SAS Institute), apart from the ICC calculations that were performed with SPSS (version 20, SPSS). Figures were made with GraphPad Prism (version 5.03, GraphPad Software). Two-sided p-values smaller than 0.05 were considered to be statistically significant.

## Results

### BI-RADS

Table 1 shows the BI-RADS density scores, as assessed by the three radiologists. Overall, 11.2% (n = 111) of the women were categorized as having ‘extremely dense’ breasts and 29.6% (n = 294) had a ‘heterogeneously dense’ breast pattern. Measures of intra-rater agreement and reliability for the BI-RADS density scores are presented in Table 1 as well. The κ_w_ ranged from 0.82 (95% CI: 0.79–0.86) to 0.87 (95% CI: 0.83–0.91). Based on the Landis and Koch guidelines (reworded by Altman), the intra-rater reliability could thus be seen as ‘very good’. The intra-rater agreement ranged from 62.8% (n = 157) to 84.8% (n = 212) (Table 1), with a mean agreement of 75.3%. When the BI-RADS scale was dichotomized (a+b vs. c+d), the proportions of agreement were larger (range %: 86.4–95.6, range n: 216–239). Only the first observer had paired scores that differed more than one category (n = 1).

**Table 1:** BI-RADS density scores: intra-rater agreement and reliability (n = 992).

	% (N)^a^			
BI-RADS density	Rater 1	Rater 2	Rater 3	Overall^b^
A	20.5 (203)	13.1 (130)	22.0 (218)	17.8 (177)
B	38.0 (377)	48.9 (485)	32.6 (323)	41.3 (410)
C	29.3 (291)	32.5 (322)	27.5 (273)	29.6 (294)
D	12.2 (121)	5.5 (55)	17.9 (178)	11.2 (111)
**Intra-rater statistics** ^c^				
% agreement	78.4 (196)	84.8 (212)	62.8 (157)	
% agreement (a+b vs. c+d)	88.4 (221)	95.6 (239)	86.4 (216)	
ᴋ_w_	0.87 (0.83–0.91)	0.85 (0.80–0.89)	0.82 (0.79–0.86)	

ᴋ_w_ = weighted kappa scores (Fleiss-Cohen, quadratic weights)

^a^ With the exception of the last row, which consists of kappa values with 95% confidence intervals.

^b^ The overall classification is based on the agreement between all three radiologists. The three radiologists all agreed in 570 of the cases. The other values were based on at least two radiologists agreeing (n = 413) or the middle value (n = 9).

^c^ The % agreement and the κ_w_ values were based on a subset (n = 250) that was scored twice by each radiologist.

All three radiologists agreed in 570 out of 992 (57.5%) assessments. Table 2 shows the inter-rater agreement and reliability for the BI-RADS density scores. The mean proportion of agreement for the pair-wise comparisons was 71.3% (range %: 67.6–74.3, range n: 671–737). The proportions were even higher when the measure was dichotomized (range %: 89.0–90.2, range n: 883–895). The κ_w_ of the inter-rater comparisons ranged from 0.80 to 0.84, which corresponds to ‘good’ or ‘very good’ reliability. In nine cases, the radiologists all scored differently. The number of discordant pairs with a difference of more than one category was limited (n = 8 for rater 1 vs. 2, n = 8 for rater 1 vs. 3, and n = 2 for rater 2 vs. 3).

**Table 2:** BI-RADS density scores: inter-rater agreement and reliability (n = 992).

		% (N)	
	ᴋ_w_ (95% CI)	% agreement	% agreement (a+b vs. c+d)
Rater 1 vs. 2	0.81 (0.78–0.83)	74.3 (737)	90.2 (895)
Rater 1 vs. 3	0.84 (0.82–0.86)	72.1 (715)	89.0 (883)
Rater 2 vs. 3	0.80 (0.78–0.82)	67.6 (671)	89.9 (892)
Rater 1 vs. majority	0.93 (0.91–0.94)	89.0 (883)	94.7 (939)
Rater 2 vs. majority	0.89 (0.87–0.91)	84.8 (841)	95.6 (948)
Rater 3 vs. majority	0.91 (0.89–0.92)	82.8 (821)	94.4 (936)

ᴋ_w_ = weighted kappa scores (Fleiss-Cohen, quadratic weights), CI = confidence interval

### Volumetric density

The volumetric breast density measures are presented in Table 3. The median volumetric breast density was 12.1% (IQR: 9.6–16.5) based on Quantra measurements, which was higher than the Volpara estimate (median: 6.6%, IQR: 4.4–10.9). Quantra also gave a higher median estimate of dense volume: 70 cm^3^ (IQR: 49–101) with Quantra compared to 50 cm^3^ (IQR: 39–70) with Volpara. Total breast volume, on the other hand, was higher for Volpara: 774 cm^3^ (IQR: 509–1119) compared to 577 cm^3^ (IQR: 368–842) for Volpara and Quantra, respectively. Based on the VDG, 12.3% and 30.7% of the women had ‘extremely dense’ (VDG 4) and ‘heterogeneously dense’ breasts (VDG 3), respectively.

**Table 3:** Volumetric breast density estimates in overall population (n = 992).

	Median (IQR)	Min	Max
**Percent dense volume (in %)**			
Volpara	6.6 (4.4–10.9)	2.0	32.1
Quantra	12.1 (9.6–16.5)	5.9	38.1
**Dense volume (in cm** ^**3**^ **)**			
Volpara	50 (39–70)	12	253
Quantra	70 (49–101)	8	403
**Total volume (in cm** ^**3**^ **)**			
Volpara	774 (509–1119)	88	3179
Quantra	577 (368–842)	59	3005
	**% (N)**		
**VDG**			
Grade 1	26.3 (261)		
Grade 2	30.6 (304)		
Grade 3	30.7 (305)		
Grade 4	12.3 (122)		

IQR = inter-quartile range

**Fig 1:**
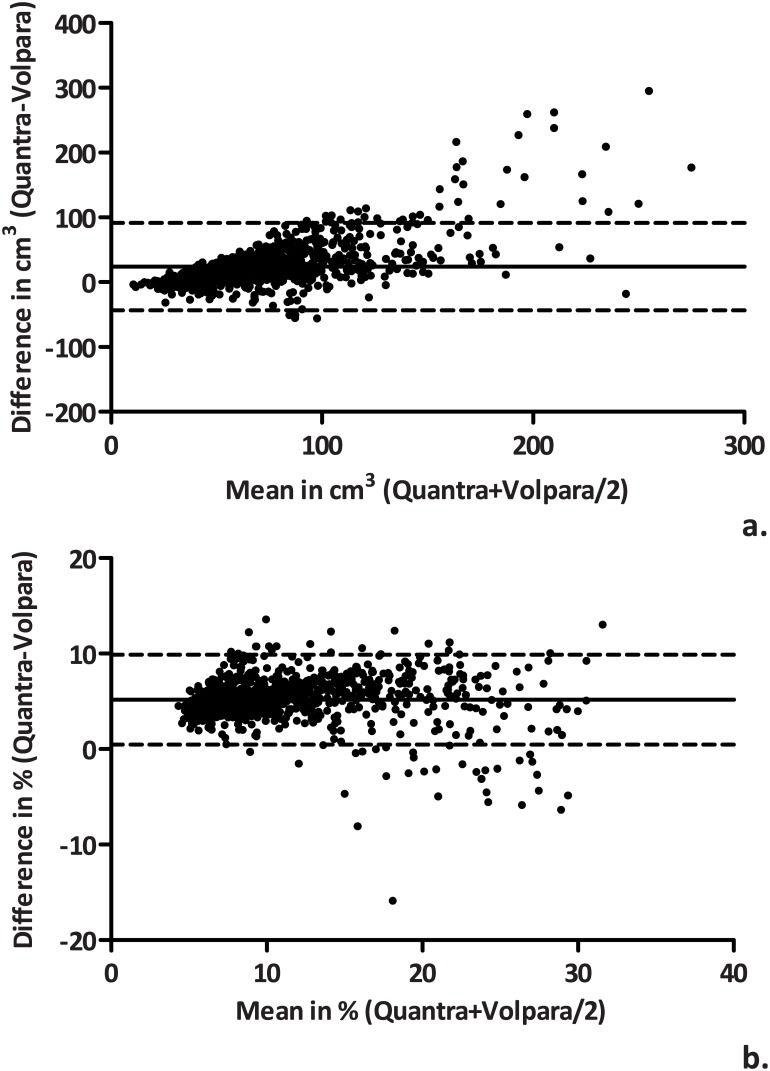
Bland-Altman plots comparing Quantra and Volpara absolute dense volume (a) and percent dense volume (b).

Fig 1 shows the agreement between the volumetric measures in Bland-Altman plots. Volpara consistently gave lower percent dense volume and absolute dense volume estimates than Quantra. The mean difference (bias) between the methods (Quantra-Volpara) was 5.19% (95% CI: 5.04–5.34) for percent dense volume and 24.1 cm^3^ (95% CI: 22.0–26.3) for dense volume. Compared with the Volpara measurement, the Quantra estimate of percent dense volume is expected to range between +0.5% and +9.9% in 95% of the measurements (limits of agreement). The Pearson’s r and the ICC were 0.91 (95% CI: 0.90–0.92) and 0.64 (95% CI: -0.07–0.88), respectively. The limits of agreement of absolute dense volume were -43.6 cm^3^ and +91.9 cm^3^, with a Pearson’s r of 0.82 (95% CI: 0.80–0.84) and an ICC of 0.55 (95% CI: 0.24–0.72).

### BI-RADS and volumetric breast density

Table 4 shows the volumetric breast density according to BI-RADS density category. For both measures, there was a clear increase in volumetric breast density as BI-RADS density increased: median estimates increased from 3.6% (IQR 3.1–4.4) to 19.3% (IQR 15.1–23.5) with Volpara and from 8.5% (IQR 7.6–9.9) to 23.1% (IQR 19.6–26.8) with Quantra. In addition, the VDG distribution was comparable to the BI-RADS density distribution (κ_w_: 0.80, 95% CI: 0.77–0.82; proportion agreement: 65.4%) (S1 Table). Volpara and Quantra did not agree on the association between BI-RADS density and absolute dense volume: a positive association was observed with Volpara, whereas Quantra estimates of absolute dense volume did not appear to be associated with the BI-RADS classification.

**Table 4:** Percent dense volume and absolute dense volume by BI-RADS density category and by age group.

		Percent dense volume						Absolute dense volume					
		Volpara			Quantra			Volpara			Quantra		
	N	Median (IQR)	Min	Max	Median (IQR)	Min	Max	Median (IQR)	Min	Max	Median (IQR)	Min	Max
**BI-RADS**													
a	177	3.6 (3.1–4.4)	2.0	9.1	8.5 (7.6–9.9)	5.9	16.8	43 (37–52)	19	130	75 (56–112)	21	403
b	410	5.3 (4.3–6.8)	3.0	15.8	10.6 (9.4–12.1)	6.6	21.4	45 (35–57)	12	124	67 (44–89)	9	277
c	294	10.2 (8.1–13.2)	4.0	26.4	16.1 (14.0–19.5)	9.5	30.1	66 (50–88)	23	189	79 (55–110)	20	364
d	111	19.3 (15.1–23.5)	7.3	32.1	23.1 (19.6–26.8)	10.2	38.1	75 (50–102)	15	253	63 (44–97)	8	290
**Age group**													
49–58	491	8.0 (4.9–13.2)	2.4	32.1	13.6 (10.3–19.0)	5.9	38.1	55 (42–78)	15	209	70 (50–105)	8	364
59–68	386	5.8 (4.2–9.5)	2.0	31.8	11.2 (9.1–15.0)	6.2	32.7	49 (37–66)	12	253	71 (49–98)	9	403
69+	115	5.4 (4.1–7.3)	2.7	19.8	10.6 (8.8–12.6)	6.6	27.0	44 (35–57)	20	134	67 (44–104)	21	272
		**BI-RADS c+d**	**BI-RADS d**		**VDG 3+4**	**VDG4**							
**Age group**		**% (N)**											
49–58	491	50.1 (246)	14.9 (73)		53.4 (262)	18.7 (92)							
59–68	386	36.0 (139)	8.8 (34)		35.8 (138)	7.0 (27)							
69+	115	17.4 (20)	3.5 (4)		23.5 (27)	2.6 (3)							

IQR = inter-quartile range

The ROC analyses on predicting the presence of BIRADS c+d (high density) with percent dense volume resulted in the following area under the curve (AUC) values: 0.948 (95% CI: 0.935–0.960) with Volpara and 0.948 (95% CI: 0.935–0.961) with Quantra (S1 Fig). The highest accuracy was observed with a cut-off value of 8.0% for Volpara (sensitivity = 84%, specificity = 91%) and 13.8% for Quantra (sensitivity = 82%, specificity = 92%).

### Age

The median age at examination was 59 years (IQR: 54–64). The median percent dense volume, Volpara and Quantra estimates, appeared to decrease with age (Table 4). The association between age and absolute dense volume was less pronounced in this population, with no clear pattern for the Quantra measurements and a slight decrease with Volpara. The percentage of women with ‘heterogeneously’ or ‘extremely’ dense breasts according to the BI-RADS density classification was lower for women in the highest age group (≥69y) compared to women in lowest age group (49–58y) (17.4% vs. 50.1%). A similar association between age and VDG was observed (23.5% vs. 53.4%).

## Discussion

We studied three different methods to assess breast density, namely the BI-RADS density scale and two software programs (Quantra and Volpara). Quantra gave higher estimates of percent dense volume and absolute dense volume than Volpara. There was a positive association between percent dense volume and the BI-RADS density scale for both programs. In addition, the VDG (Volpara measure) seemed to be a good approximation of BI-RADS density in our study. Absolute dense volume only appeared to be associated with BI-RADS density when using the Volpara estimates. These density measures may potentially be used in the evaluation of screening performance and to identify risk groups.

Although other studies used older editions of the BI-RADS classification, the intra- and inter-observer reliability estimates in our study appeared to be similar to previous findings [10, 28–32]. The κ_w_ tends to suggest ‘good’ to ‘very good’ reliability based on the Landis and Koch guidelines, even though these categories may be somewhat arbitrary. The proportions of agreement improved after the consensus meeting (data not shown), but there are still relatively large discrepancies between the radiologists (up to 32.4% for observer 2 vs. 3). For this reason, density assessment by individual radiologists is not useful for selecting women for future alternative screening regimens in population-based organised breast cancer screening programmes or risk management. Furthermore, the intra- and inter-rater variability may differ between radiologists, for example based on experience level [33].

The use of automated volumetric density measures has been advocated [19, 34]. Volumetric density would have several advantages over qualitative scales and area-based density measures. Volumetric software programs calculate breast density based on 3D instead of 2D information, thus also including thickness of the tissue. An estimate of the actual volume of the tissue rather than the 2D projection of the tissue is expected to have a stronger biological association [12, 13]. In addition, the calculations incorporate imaging settings (e.g., X-ray dose). Furthermore, with both software programs there is perfect agreement between two assessments of the same mammogram, which we also observed in our data. This is in contrast to the qualitative and semi-automated measurements, in which some degree of intra- and inter-rater variation appears to be inevitable. Finally, the volumetric measurements would be easier to implement in screening programmes as the automated software tends to be less time-consuming and labour-intensive than the rather variable visual assessment with BI-RADS breast density, which in dual reading set-up will cause many discrepancies.

Several studies have compared the volumetric estimates to the BI-RADS scale (Table 5) [35–41]. An important difference between radiologists’ scores and automated methods is that radiologists tend to give the maximum value (as suggested by the ACR), whereas volumetric density estimates are based on the average of multiple views. The results from all these studies do, however, suggest a clear positive association between percent dense volume and BI-RADS density. The median estimates of percent dense volume we obtained with Volpara for each BI-RADS category appeared to be at the lower end of the range. Our Quantra estimates were lower than the available literature values as well. This may be explained by differences in setting and risk factor distribution (e.g., age range, use of hormone therapy, clinic versus screening). Using area-based measures, the highest BI-RADS density category was previously linked to density percentages greater than 75% (4^th^ BI-RADS edition). All our volumetric estimates for percent dense volume were below 40%, which clearly illustrates a difference in range between area-based and volumetric methods. Similar to our findings, Gweon *et al*. and Jeffreys *et al*. both found an increase in absolute dense volume with increasing BI-RADS density [35, 36]. We observed a distinct difference in Volpara absolute dense volume between the two lowest and the two highest BI-RADS density categories. There was no clear association between Quantra absolute dense volume and BI-RADS density. In line with these results, Eng *et al*. found that Quantra absolute dense volume, in contrast to Volpara dense volume or Cumulus dense area, was not associated with an increased breast cancer risk (Q5 vs. Q1: OR 1.08) [42].

**Table 5:** Association between BI-RADS density measures and volumetric density in other studies.^a^

		BI-RADS 1/a	BI-RADS 2/b	BI-RADS 3/c	BI-RADS 4/d	Association measures
**Volpara: percent dense volume**	**N**	**Mean (SD)**				
Gweon [35]	778	6.1 (0.9)	7.8 (2.3)	14.1 (5.8)	26.1 (5.2)	% density vs. BI-RADS (Spearman’s ρ) = 0.765; VDG vs. BI-RADS (κ_w_) = 0.54
	**N**	**Median (IQR)**				
Current study	992	3.6 (3.1–4.4)	5.3 (4.3–6.8)	10.2 (8.1–13.2)	19.3 (15.1–23.5)	
Jeffreys [36]	324	4 *(3–5)*	*6 (4–8)*	*11 (8–16)*	18.9 *(15–22)*	
Seo [37]	193	*5 (4–8)*	*9 (7–11)*	*14 (12–17)*	*21 (18–25)*	% density vs. BI-RADS (Spearman’s ρ) = 0.754; VDG vs. BI-RADS (ICC) = 0.757
Gubern-Mérida [38]	186	5.66 *(5–6)*	*9 (8–11)*	*21 (18–24)*	26.69 *(24–29)*	% density vs. BI-RADS (Spearman’s ρ) = 0.79; VDG vs. BI-RADS (κ_w_) = 0.40
Ko [39]	1129					VDG vs. BI-RADS (κ) = 0.26
**Volpara: dense volume**	**N**	**Mean (SD)**				
Gweon [35]	778	33.7 (7.3)	40.7 (13.3)	50.7 (23.7)	63.8 (35.4)	
	**N**	**Median (IQR)**				
Current study	992	43 (37–52)	45 (35–57)	66 (50–88)	75 (50–102)	
Jeffreys^b^ [36]	324	38 *(30–50)*	*50 (40–60)*	*60 (50–80)*	80 *(60–120)*	
**Quantra: percent dense volume**	**N**	**Mean (SD)**				
Regini [40]	200	13.4 (3.6)	16.8 (4.2)	24.5 (7.2)	32.8 (8.3)	% density vs. BI-RADS (Pearson’s r) = 0.99; Cut-off value BI-RADS3+4 = 21%
	**N**	**Median (IQR)**				
Current study	992	8.5 (7.6–9.9)	11.0 (9.4–12.1)	16.1 (14.0–19.5)	23.1 (19.6–26.8)	
	**N**	**Median (range)**				
Ciatto^c^ [41]	418	12.0 (7.0–19.0)	17.5 (10.0–32.0)	27.5 (16.0–47.0)	33.0 (24.5–50.5)	Cut-off value BI-RADS3+4 = 22%
**Quantra: dense volume**	**N**	**Median (IQR)**				
Current study	992	75 (56–112)	67 (44–89)	79 (55–110)	63 (44–97)	

SD = standard deviation, IQR = inter-quartile range

^a^ The results in italics were not published, but were based on estimates from the published boxplots.

^b^ Values were rounded off.

^c^ Also presented means, which were: 12.2 for BI-RADS 1, 18.0 for BI-RADS 2, 28.5 for BI-RADS 3, and 34.5 for BI-RADS 4.

There was a relatively strong correlation in percent dense volume between the two automated volumetric methods (Pearson’s r: 0.91, ICC: 0.64). The correlation for absolute dense volume, on the other hand, appeared to be somewhat weaker, with lower correlation coefficients (Pearson’s r: 0.82, ICC: 0.55). The first results from validation studies, comparing volumetric density to MRI results, are now appearing in the literature. Gubern-Mérida *et al*. indicated that Volpara may slightly underestimate the true density (as measured with MRI) [38]. Wang *et al*. is, to our knowledge, the first study to include both Volpara and Quantra. They observed a strong correlation between the two measures, as well as a strong correlation of both with MRI [14]. However, absolute dense volume was not included in either of these studies. Morrish *et al*. did report on absolute dense volume in their comparison study of Quantra and Volpara [15]. Although they observed a weaker correlation for percent dense volume, the results on absolute dense volume appear to be in line with our findings. It should be noted that this study was performed in a slightly different setting (e.g., country, age range, participant selection) and used different software versions, which may explain differences in volume estimates and observed correlations.

The effect of breast density on breast cancer risk is relevant for personalised (primary and secondary) prevention, where it can potentially be used as a risk stratification factor. Little evidence has yet been published on the association between volumetric density and breast cancer risk to date, although previous studies have suggested that volumetric density may be more strongly associated with breast cancer risk due to its predicted biological association [12, 13]. According to the meta-analysis of McCormack *et al*. [3], women with extremely dense breasts based on the BI-RADS classification have a 4.08 (95% CI: 2.96–5.63) times higher breast cancer risk compared with women with fatty breasts. In our study, the highest BI-RADS category corresponded to a median percent dense volume of 19.3% (Volpara) or 23.1% (Quantra). However, with overlapping ranges of volumetric density for different BI-RADS categories, it is difficult to directly relate these findings to the previously determined risks based on the BI-RADS scale. Park and colleagues reported an adjusted OR of 3.07 for women with more than 15.1% Volpara percent dense volume compared to women with less than 4.7% in a Korean population [43]. A study by Brand *et al*. showed that the highest Volpara density quartile was associated with a 2.93 (percent dense volume) or 1.63 (absolute dense volume) higher risk than the lowest quartile [44]. Finally, Eng *et al*. studied several breast density measures: both Volpara (Q5 vs. Q1: OR 8.26) and Quantra (Q5 vs. Q1: OR 3.94) estimates of percent dense volume were associated with an increased breast cancer risk [42].

The associations between volumetric density and other established breast cancer risk factors may provide some insight into the etiological role of volumetric density. We studied the association with age, where we observed a similar inverse association as has previously been determined using other density measures. Studies have shown that most risk factors have a similar association with Volpara volumetric breast density as they do with area-based measures [44–46]. Only limited evidence is available on the association between established risk factors and Quantra volumetric density [47].

One of the limitations of our study is that we did not have any information on breast cancer risk, which would ultimately be needed to validate both breast density measures and potentially implement them in a breast cancer screening setting if they are to be used for risk stratification. More research is needed as well on the association between volumetric density and sensitivity of digital mammography. This information is required to identify a clinically relevant breast density cut-off value above which additional screening (e.g., with MRI or ultrasound) may be cost effective. Studies are also needed on the potential inclusion of volumetric density in risk models. Strengths of the current study include the use of both Volpara and Quantra, which we were able to study in relation to the newest BI-RADS density classification. In addition, we included both percent dense volume and absolute dense volume. Finally, our study sample was relatively large compared to previous studies (Table 5).

Before volumetric density measurements can be implemented in breast cancer screening, the infrastructure on storing unprocessed mammogram data has to be developed further. This would involve large amounts of data. However, the advantage of this data storage is that multiple automated tools can easily be compared over time. Furthermore, if at any time an algorithm would be introduced that performs considerably better, it could also be applied to historical data. This is especially important for monitoring density changes, for example between geographic areas and within women. Due to the lack of intra- and inter-observer variability, in contrast to the BI-RADS density classification, changes in density can be more readily detected if random measurement error is small.

## Conclusions

Volpara and Quantra clearly differed from each other. However, there appeared to be a strong association of these measures with each other and with the BI-RADS density scale. Further research on the differences between the measures is needed before they can be implemented in breast cancer screening programmes. This applies both to the logistics surrounding breast density measurements and the role of breast density in screening programmes. If studies indeed show that breast density is important for evaluating performance or could be useful for risk stratification, then both Quantra and Volpara may be considered.

## Supporting Information

S1 Fig ROC analyses on predicting high density (BI-RADS c+d) with percent dense volume (a) and dense volume (b). (PDF)

S1 Table Comparison of VDG and BI-RADS density classification (N, %). (PDF)
