# Supplementary material for: Comparing Visually Assessed BI-RADS Breast Density and Automated Volumetric Breast Density Software: A Cross-Sectional Study in a Breast Cancer Screening Setting
Source: PLoS One. 2015 Sep 3;10(9):e0136667. doi: 10.1371/journal.pone.0136667 (PMC4559403; doi:10.1371/journal.pone.0136667)
Supplement: S1 Fig — (PDF) [file pone.0136667.s001.pdf]

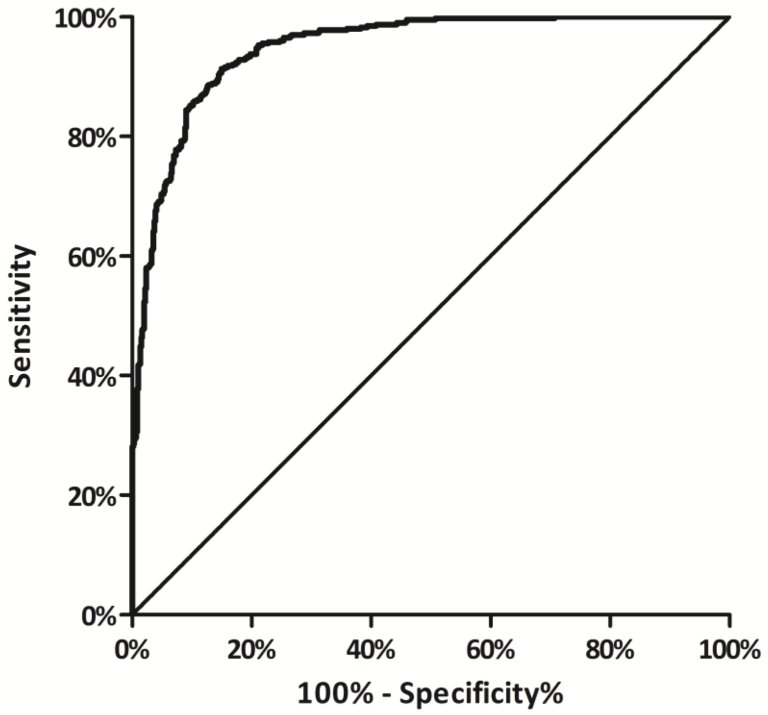

a.

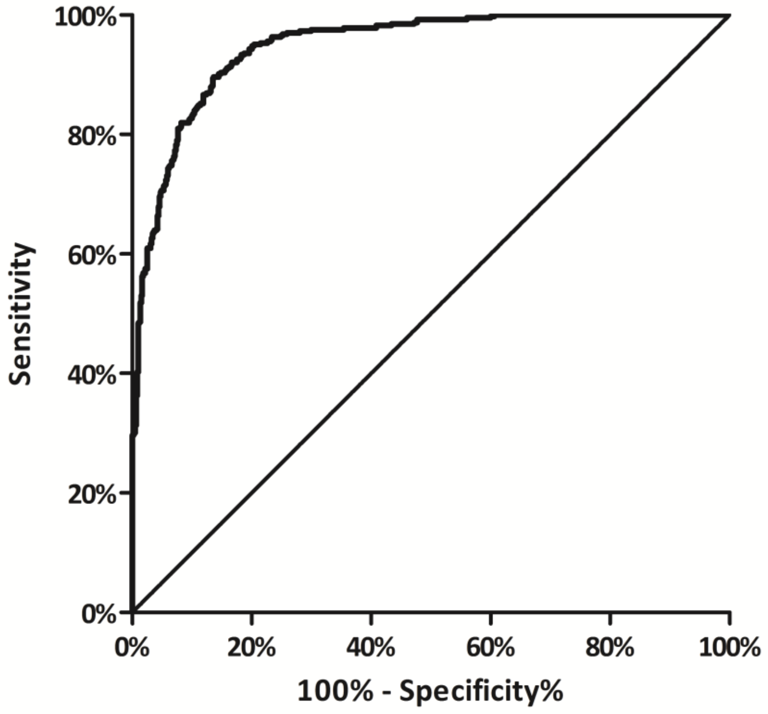

b.

Supplementary figure 1 – ROC analyses on predicting high density (BI-RADS c+d) with percent dense volume (a) and absolute dense volume (b)
