# Supplementary material for: Comparing Visually Assessed BI-RADS Breast Density and Automated Volumetric Breast Density Software: A Cross-Sectional Study in a Breast Cancer Screening Setting
Source: PLoS One. 2015 Sep 3;10(9):e0136667. doi: 10.1371/journal.pone.0136667 (PMC4559403; doi:10.1371/journal.pone.0136667)
Supplement: S1 Table — (PDF) [file pone.0136667.s002.pdf]

Supplementary table 1 - Comparison of VDG and BI-RADS density classification (N, %)

	VDG1	VDG2	VDG3	VDG4	Total
**BI-RADS a**	140 (14.1)	36 (3.6)	1 (0.1)	0 (0.0)	177 (17.8)
**BI-RADS b**	120 (12.1)	218 (22.0)	69 (7.0)	3 (0.3)	410 (41.3)
**BI-RADS c**	1 (0.1)	49 (4.9)	208 (21.0)	36 (3.6)	294 (29.6)
**BI-RADS d**	0 (0.0)	1 (0.1)	27 (2.7)	83 (8.4)	111 (11.2)
**Total**	261 (26.3)	304 (30.6)	305 (30.7)	122 (12.3)	992 (100.0)
